# Swift Large-scale Examination of Directed Genome Editing

**DOI:** 10.1371/journal.pone.0213317

**Published:** 2019-03-05

**Authors:** Omar T. Hammouda, Frank Böttger, Joachim Wittbrodt, Thomas Thumberger

**Affiliations:** 1 Centre for Organismal Studies Heidelberg, Heidelberg University, Heidelberg, Germany; 2 Heidelberg Biosciences International Graduate School, Heidelberg University, Heidelberg, Germany; University Zürich, SWITZERLAND

## Abstract

In the era of CRISPR gene editing and genetic screening, there is an increasing demand for quick and reliable nucleic acid extraction pipelines for rapid genotyping of large and diverse sample sets. Despite continuous improvements of current workflows, the handling-time and material costs per sample remain major limiting factors. Here we present a robust method for low-cost DIY-pipet tips addressing these needs; i.e. using a cellulose filter disc inserted into a regular pipet tip. These filter-in-tips allow for a rapid, stand-alone four-step genotyping workflow by simply binding the DNA contained in the primary lysate to the cellulose filter, washing it in water and eluting it directly into the buffer for the downstream application (e.g. PCR). This drastically cuts down processing time to maximum 30 seconds per sample, with the potential for parallelizing and automation. We show the ease and sensitivity of our procedure by genotyping genetically modified medaka (*Oryzias latipes*) and zebrafish (*Danio rerio*) embryos (targeted by CRISPR/Cas9 knock-out and knock-in) in a 96-well plate format. The robust isolation and detection of multiple alleles of various abundancies in a mosaic genetic background allows phenotype-genotype correlation already in the injected generation, demonstrating the reliability and sensitivity of the protocol. Our method is applicable across kingdoms to samples ranging from cells to tissues i. e. plant seedlings, adult flies, mouse cell culture and tissue as well as adult fish fin-clips.

## Introduction

Gene-editing tools such as CRISPR/Cas9 have revolutionized genome editing in most model organisms [1–3]. The easy application of this targeting approach in organisms as well as cell culture is driving extensive high-throughput applications such as genetic screens [4,5]. Common to all genome targeting applications is the need for rapid subsequent genotyping. Following PCR amplification of the targeted loci, among the most commonly used assays for indel detection are mismatch cleavage assays (using T7 Endonuclease I or Cel-based Surveyor assay), high-resolution melting analysis (HRMA) and sequencing [6,7] in combination with Tracking of Indels by Decomposition (TIDE, [8]). However, the high-throughput approaches face a bottle neck when it comes to genomic DNA (gDNA) extraction, which usually requires a time- and material-consuming protocol or is not immediately applicable to various sample material, e.g. medaka embryos.

Genome extraction has greatly improved due to the numerous advances on nucleic acid purification methods. These either rely on solid-phase extraction kits or on traditional phenol-chloroform purification, both of which are time- or material-consuming and require a lot of steps [9]. Other more straight-forward protocols like alkaline lysis rely on chemical disintegration of tissues by (extended) incubation steps followed by neutralization [10]. Alternatively, incubation can be avoided using membranes of different materials to bind nucleic acids with high affinity, eliminating the need for nucleic acid purification by direct amplification of the nucleic acid off the membrane [11–14]. A recent improvement has greatly reduced the genome extraction step to under 30 seconds using cellulose paper-based dipsticks [15]. Although extremely advantageous in terms of time and material, these methods require fabrication or manufacturing steps, which make them unsuitable for high-throughput applications, especially for automated systems.

Here we present a genotyping protocol, which has great potential for high-throughput and automated applications. Our four-step procedure breaks the cells open and binds nucleic acids to cellulose. The bound nucleic acids are washed and eventually eluted directly with the buffer of the downstream application. The rapid extraction of nucleic acids is facilitated by inserting cellulose paper discs into conventional pipet tips (filter-in-tips). We validated the approach by rapid genotyping of various CRISPR/Cas9 edited medaka and zebrafish embryos in a 96 well-based format. To parallelize lysis of 96 samples, we have devised a custom-made mortar (‘hammer’). Subsequent use of a multichannel pipet equipped with filter-in-tips, allows the Swift Large-scale Examination of Directed Genome Editing (SLEDGE hammer). The filter-in-tips approach is widely applicable whenever rapid isolation of nucleic acids is aimed for as shown by gDNA extraction from varying sources of tissues derived from diverse (model) organisms such as plants (*Arabidopsis thaliana*), insects (*Chironomus riparius* and *Drosophila melanogaster*), fish (medaka, *Oryzias latipes*; zebrafish, *Danio rerio*) embryos and fin-clips as well as mouse (*Mus musculus*) ear punches and embryonic stem cells.

## Results and discussion

Teleost fish have been extensively used for high-throughput approaches [16–19]. The recent advances in CRISPR/Cas9 mediated genome editing [3,20] as well as the establishment of the medaka inbred panel [21] as a population genomic resource have led to rising demand on high throughput genotyping for phenotype-genotype correlation.

In light of the reported nucleic acid binding property of cellulose based filter paper [15], we sought to exploit this feature to by-pass incubation and/or additional purification steps of established gDNA extraction methods.

**Fig 1:**
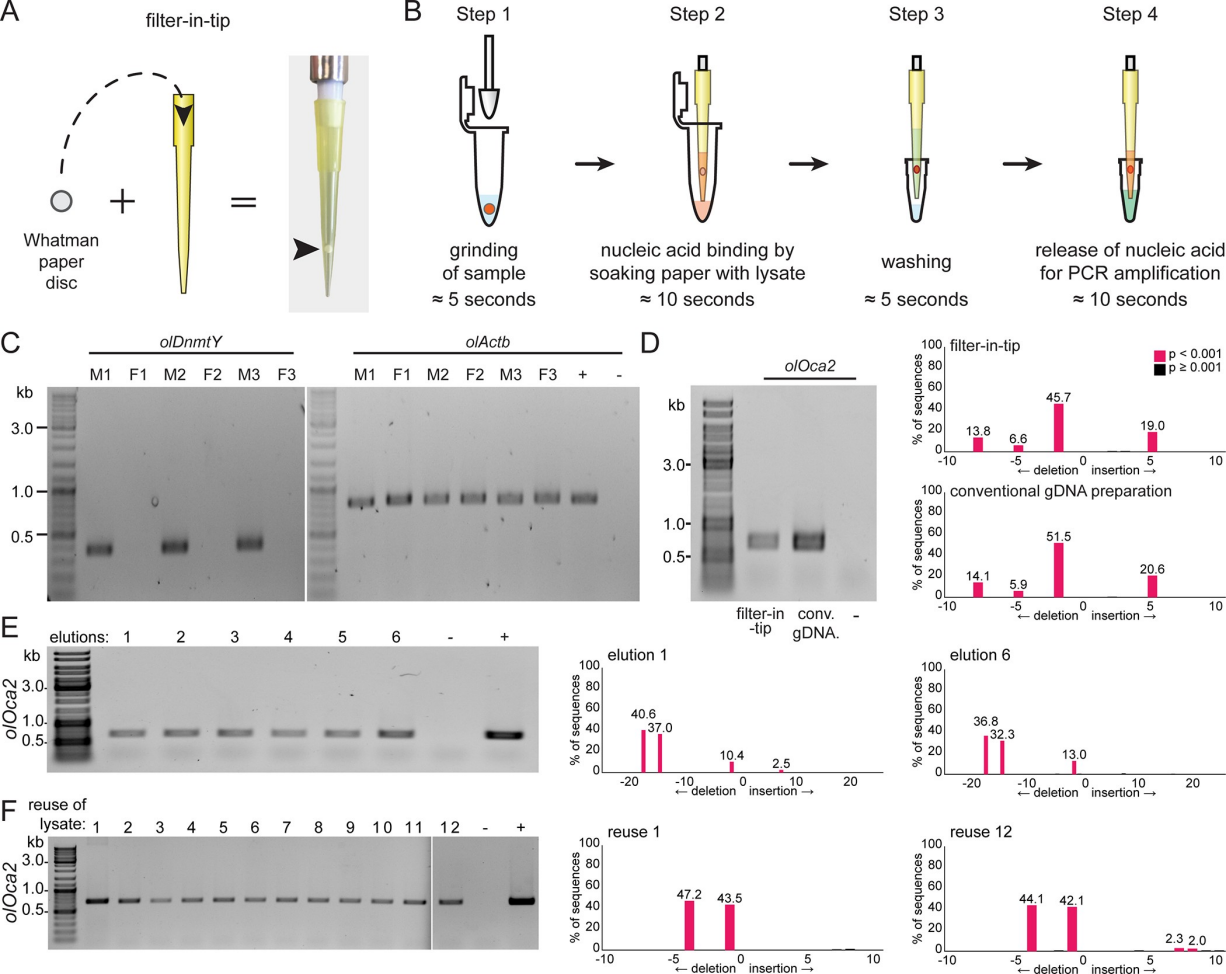
Filter-in-tips are highly sensitive for rapid transfer of nucleic acids for targeted genome editing diagnostics. A) Assembly of filter-in-tip–Whatman filter paper / cellulose disc inserted into 200 μl yellow tip at the sample-proximal end. B) Schematic workflow of nucleic acid extraction using filter-in-tip. Step 1 lysis: pestle used to grind sample (red) in fin-clip buffer. Step 2 binding: using a filter-in-tip, pipet up lysate to let nucleic acids (red) bind to cellulose filter disc (soak for ≈10 sec). Release lysate back into tube for storage. Step 3 washing: wash filter disc containing nucleic acids (red) by pipetting nuclease free water in and out (≈5 sec). Step 4 elution: pipet up pre-mixed PCR mixture (wait ≈10 sec) to release nucleic acids and pipet back for amplification. C) Genomic male sex determination by *olDnmtY* PCR of 3 male (M1-3) and 3 female (F1-3) medaka fin clips. *olActb* amplification as controlNote: transfer of gDNA via filter-in-tip and amplification of loci was successful in all cases. D) Comparison between gDNA extracted with filter-in-tip (left) versus conventional gDNA preparation (right) of a single stage 32 medaka *oca2* crispant. Note: matching allele distribution profiles of both amplicons (TIDE analysis). E) DNA retention capacity of a single filter-in-tip: cellulose filter soaked only once with a stage 32 medaka *oca2* crispant lysate (10 seconds), washed once and eluted 6 individual times (10 seconds each). Note: matching allele distribution profiles of first and last amplicon (TIDE analysis). F) Repeated extraction of gDNA from a single stage 32 medaka *oca2* crispant lysate using 12 filter-in-tips. Note: matching allele distribution profiles of first and last amplicon (TIDE analysis). wt gDNA (conventional gDNA preparation) as positive control (+), water as negative control (-).

We established the filter-in-tip as a high-throughput nucleic acid extraction tool. We used a paper puncher to cut Whatman filter paper discs (≈2 mm) and placed them inside of standard yellow 200 μl pipet tips (Fig 1A). We tested the applicability of these modified pipet tips for rapid genotyping. Fins of three male and three female wild-type medaka adults were clipped and administered for gDNA extraction and subsequent use for determination of the genetic sex by PCR. Fin clips were put into 100 μl fin-clip buffer-containing Eppendorf tubes. After grinding the tissue with individual pestles (Fig 1B, step 1), the gDNA lysate was pipetted into filter-in-tips. Letting the lysate rest in the tip for ≈10 seconds allowed the Whatman paper disc to bind the nucleic acids (Fig 1B, step 2). The lysate was released back into the tube for storage. Next, the filter was washed to remove the lysis buffer (potentially impairing later diagnostics) by a brief pipetting step in Milli-Q water (Fig 1B, step 3). For elution, the PCR reaction mix was pipetted into the very same filter-in-tip. Letting it rest for ≈10 seconds released bound nucleic acids from the Whatman paper. Finally, the reaction mix was pipetted back into the PCR-tube for immediate amplification (Fig 1B, step 4).

The approach was successful for all specimens. The *olDnmtY* band correlated with the male sex phenotype and the *olActb* control amplicon was present in all extracted gDNAs (Fig 1C). To compare the quality and composition of gDNAs prepared by either the filter-in-tip or conventional gDNA extraction approach (Blin-Stafford lysis followed by ethanol precipitation [22]), we turned to genetically mosaic medaka embryos resulting from a CRISPR/Cas9 induced mutagenesis (so-called crispants [23]) in the *oculocutaneous albinism 2* locus (*oca2* [24]). Each of those embryos contains a specific mix of mutant alleles of different abundancies. A single embryo (stage 32) was lysed and gDNA was bound and processed following the filter-in-tip approach described above. The remainder of the lysate was processed following the conventional gDNA extraction protocol. Both approaches resulted in clearly detectable PCR products with matching allele distribution profiles as determined by Sanger sequencing and downstream TIDE analysis of the targeted *oca2* locus (Fig 1D). This illustrates a high quality of the filter-in-tip extracted gDNA.

We next addressed the DNA retention capacity of the filter disc. A single filter-in-tip was loaded once (10 seconds) with a stage 32 *oca2* crispant embryo lysate, washed once and eluted six times sequentially (10 seconds each) to result in six separate PCR mixes. *Oca2* locus amplification was successful in all six eluates with a matching allele distribution profile (TIDE analysis) between the first and sixth eluate (Fig 1E). This indicates a high gDNA binding capacity of the filter-in-tip, sufficient for at least six eluates of gDNA. The gDNA is released in an unbiased manner, preserving the complex allele structure.

Finally, we addressed the reproducibility of our filter-in-tip approach by sequentially extracting gDNA from the lysate of a single *oca2* crispant embryo (stage 32) using twelve filter-in-tips. PCR amplification of gDNA eluted from each filter-in-tip successfully yielded a PCR product. TIDE analysis of the first and last PCR amplicons showed matching allele distribution profiles (Fig 1F).

In a conservative estimate (assuming four successful elutions from a single bound filter-in-tip and twelve bindings per lysed embryo) the presented filter-in-tip approach allows the extraction of DNA sufficient for at least 48 individual PCR reactions from a single medaka embryo (stage 32), which at least matches the yield of alternative protocols.

**Fig 2:**
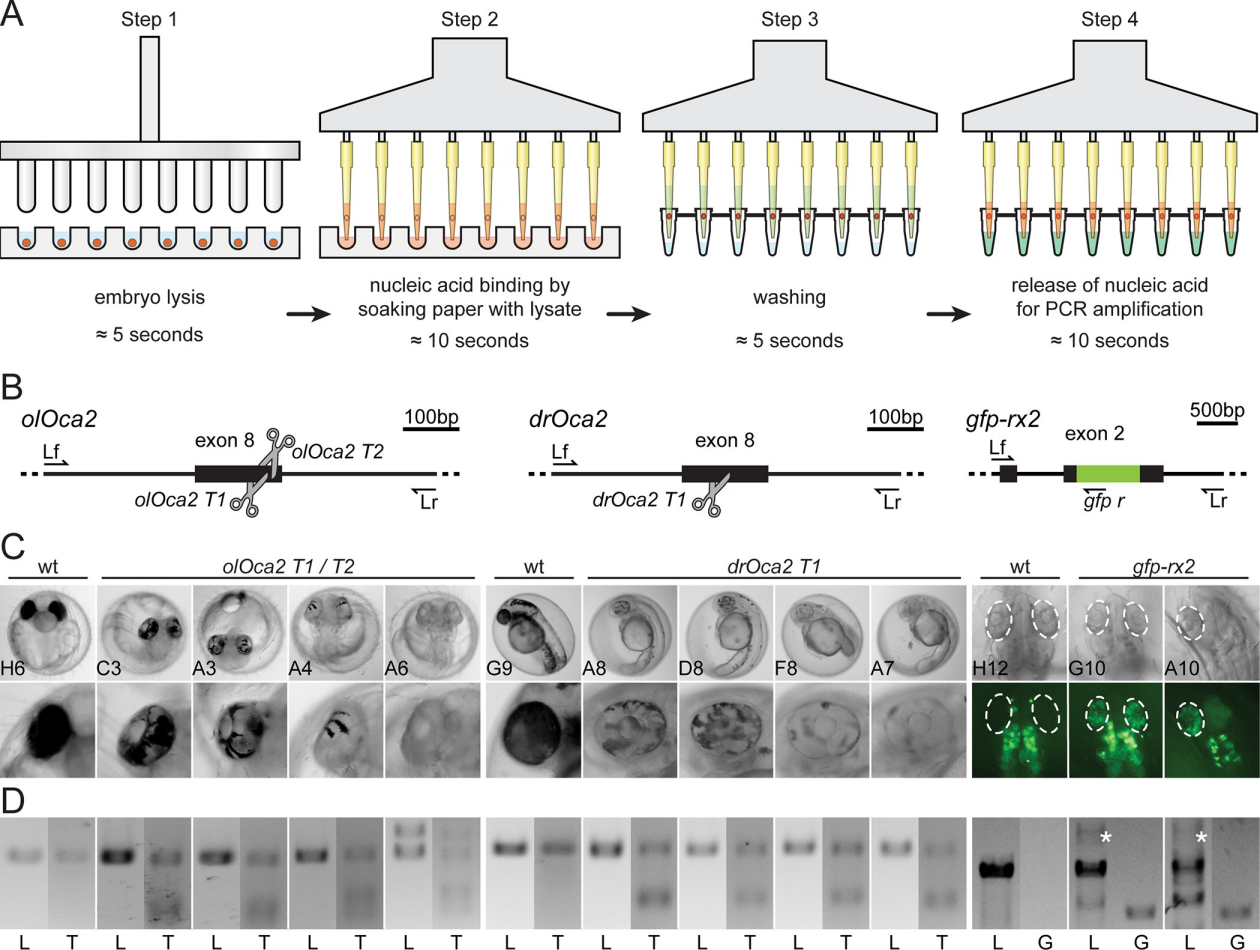
Application of filter-in-tips in high throughput, SLEDGE hammer protocol. A) Schematic workflow of high-throughput 96-well plate based SLEDGE hammer protocol with filter-in-tips. Step 1 lysis: (96-pin) mortar used to simultaneously grind individual embryos in fin-clip buffer. Step 2 binding: using a multichannel pipet equipped with filter-in-tips, pipet up lysate to let nucleic acids (red) bind to cellulose filter discs (soak for ≈10 sec). Release lysate back into wells for storage. Step 3 washing: wash filter discs containing nucleic acids (red) by pipetting nuclease free water in and out (≈5 sec). Step 4 elution: pipet up pre-mixed PCR mixture (wait ≈10 sec) to release nucleic acids and pipet back for amplification. Note: entire procedure takes less than 10 minutes (6 minutes processing time). B) Schematic representation of CRISPR/Cas9 mediated NHEJ-based knock-out (medaka *oca2*, *olOca2*; zebrafish *oca2*, *drOca2*) and HDR-mediated single-copy integration of *gfp* sequence in frame with medaka *rx2* (cf. Gutierrez et al., 2018 [20]). Location of sgRNA target sites shown by scissors. Primers used for analysis indicated; locus forward (Lf), locus reverse (Lr) of the respective gene. *rx2* locus shown after single-copy HDR-mediated integration of *gfp*. C) Besides fully pigmented eyes in wild-type (wt) medaka and zebrafish, varying degrees of pigment loss in body and RPE (blow-up) of representative crispants injected with individual sgRNAs targeting *oca2* exon 8. Retinae (dashed ellipses) of injected embryos show GFP expressing cells after HDR/donor mediated integration of *gfp* into *rx2* open reading frame. Note: unspecific autofluorescence of body pigment. 96-well plate coordinates of specimens indicated (see S2 Fig). D) Genotyping of representative embryos in C. Locus PCR (L; primers Lf/Lr) of respective genes and T7EI assay (T) validating indel formation in *oca2* loci. In *gfp-rx2* tagging, besides non-*gfp*-integrated locus band (L; primers Lf/Lr), single *gfp* integration evident by PCR (white asterisk). Integration validated by locus-gfp band (G; primers Lf/*gfp* r).

We next expanded the range of this protocol to large scale individual extractions of gDNA. To achieve this, we combined the parallel lysis of specimens in a 96 well plate using a custom-made 96 pin mortar, ‘the hammer’ (see S1 Fig for plan and materials and methods section for detailed instructions), with parallel processing of filter-in-tips by a multichannel pipettor (Fig 2A).

To test whether the extracted gDNA allows a direct phenotype-genotype correlation we used different embryos (medaka, zebrafish) in which different CRISPR/Cas9 experiments had been performed (*oca2* knock-out [24], *Rx2* GFP knock-in [20]; Fig 2B) that all result in a scorable phenotype.

Per *oca2* sgRNA, 21 injected embryos (4 days post fertilization (dpf) for medaka, 2 dpf for zebrafish) were randomly selected and put into individual wells of a 96-well U-shaped microtiter plate for phenotype/genotype correlation. For screening of *gfp-rx2* integration, 21 injected medaka embryos were randomly picked at 2 dpf and placed to individual wells of the same plate (S2 Fig). One negative control (empty well containing embryo rearing medium) as well as two non-injected control embryos for each species and stage complemented each group.

Phenotyping revealed that the targeted inactivation of the *oca2* gene resulted in the expected loss of pigmentation in all specimens with various extents as evident in the retinal pigmented epithelium (RPE; Fig 2C). Tagging of the *rx2* gene was visible by specific GFP expression in the medaka retinae (Fig 2C). HDR-mediated knock-in attempts are less efficient than knock-out approaches, still ≈40% (8/21) of injected specimens showed varying numbers of GFP positive cells in the developing eye. To correlate with the observed phenotype, each embryo was genotyped individually.

After phenotyping (plate imaging), the fish medium in the 96 U-well plate was replaced with fin-clip lysis buffer (100 μl per well) and the embryos were simultaneously lysed by single push down of the “hammer” (96-well mortar). We followed the rapid gDNA extraction approach described above using the filter-in-tips in combination with a multichannel pipet (Fig 2A). This entire procedure took less than 10 minutes (6 minutes processing time).

For the *oca2* knock-out embryos, locus amplification revealed that in all cases transfer of gDNA to the PCR mixture was successful (Fig 2D, S2 Fig). We subsequently examined the success of targeted genome editing (indel formation) in the *oca2* loci using T7 Endonuclease I (T7EI) assay, which detects and cuts heteroduplexes caused by a heterogeneous mixture of indel alleles from genome targeted specimens. T7EI genotyping confirmed the phenotypic observation, i.e. all injected embryos of all *oca2* targeting attempts showed heteroduplex digestion in the T7EI assay, whereas the locus amplicon of the respective non-injected controls did not (Fig 2D, S2 Fig).

Furthermore, all *gfp-rx2* tagging approaches that were positive in the GFP screening could as well be confirmed by PCR genotyping as fusion of the *gfp* sequence with the *rx2* locus in the respective specimens (Fig 2D, S2 Fig). It is interesting to note that our approach allowed to distinguish single-copy HDR-mediated integration (7 out of the 8 positively screened embryos, Fig 2D, S2 Fig) and NHEJ-mediated concatenation and integration (S2 Fig).

Genotyping of the entire 96-well plate without losing a single specimen highlights that the filter-in-tip approach is robust, reliable and reproducible. The genotype/phenotype correlation further demonstrates the approach to be accurate and sensitive, reflecting the homogeneous extraction of delicate gDNA samples.

**Fig 3:**
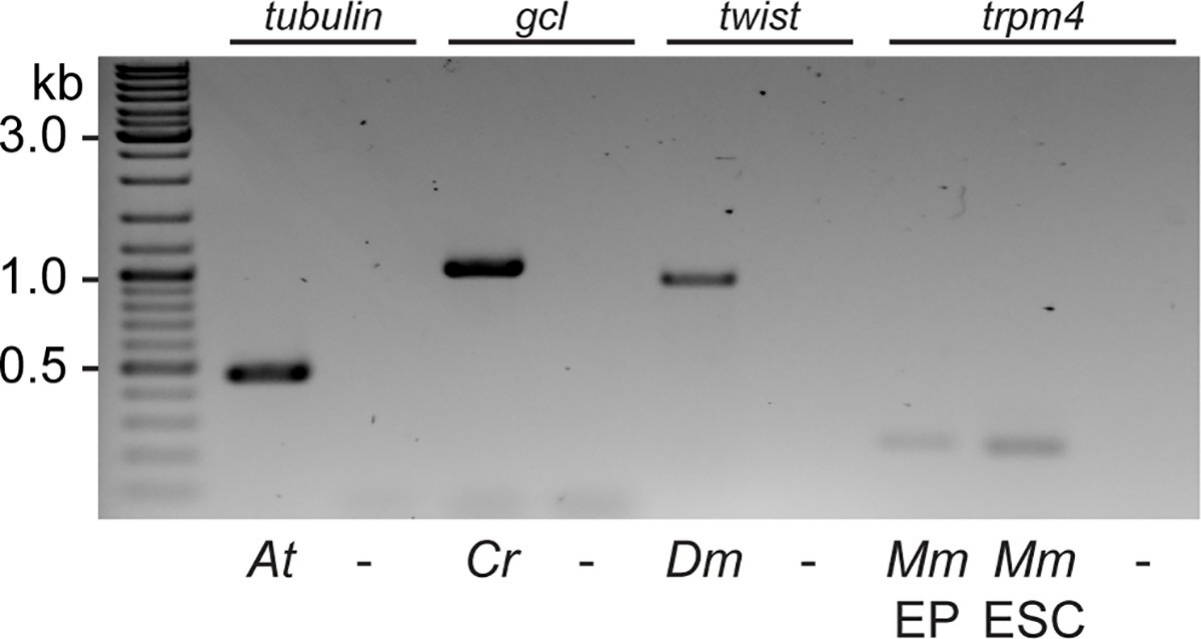
General applicability of filter-in-tips to rapidly transfer gDNA from various sources. Filter-in-tips successfully transferred gDNA from lysates of *Arabidopsis thaliana* (*At*), *Chironomus riparius* (*Cr*), *Drosophila melanogaster* (*Dm*), mouse (*Mus musculus*, *Mm*) ear punches (EP) and mouse embryonic stem cells (ESCs) as evident by PCR amplification of candidate genes (*tubulin*; *germ cell-less*, *gcl*; *twist*; *trpm4*). Water control (-).

Besides the reliable extraction of gDNA from fish embryos and tissues, we investigated if this approach is of general use for gDNA extraction from varying sources of tissues derived from (model) organisms such as *Arabidopsis thaliana*, *Drosophila melanogaster*, *Chironomous riparius*, mouse ear punches (EP) and mouse embryonic stem cells (mESC) (Fig 3). In all cases the filter-in-tips approach yielded sufficient material for specific locus PCR analyses. Extraction was performed as described above. Tissue was lysed by grinding with the “hammer”, i.e. a single seedling of Arabidopsis, an individual adult fly of either insect species, an individual mouse ear punch or 2.5 x 10^5^ ESC suspension in lysis buffer. From the lysate gDNA of the different origins was transferred by pipetting of the lysate using our filter-in-tips and eventually eluted in the respective PCR mix for specific locus amplification. The rapid lysis, transfer, binding and elution resulted in sufficient material for the downstream analysis of all species and tissues or cells tested so far.

Taken together, our SLEDGE hammer protocol with the filter-in-tips allows to bypass incubation/purification steps of conventional gDNA preparation protocols. With the rapid genotyping method presented here, future attempts of individual phenotype-genotype correlation following high-throughput automated screening of individual specimens are in reach. Furthermore, our filter-in-tips can easily be applied to fully automated applications for any DNA containing sample rendering it a fast and straight-forward application for any routine genotyping.

## Materials and methods

### Fish husbandry and ethics statement

All fish are maintained in closed stocks at Heidelberg University. Medaka (*Oryzias latipes*) and zebrafish (*Danio rerio*) husbandry (permit number 35–9185.64/BH Wittbrodt, Regierungspräsidium Karlsruhe) were performed according to local animal welfare standards (Tierschutzgesetz §11, Abs. 1, Nr. 1) in accordance with European Union animal welfare guidelines [25]. The fish facility is under the supervision of the local representative of the animal welfare agency. Embryos of medaka of the wildtype Cab strain and of zebrafish AB/AB line were used at stages prior to hatching. Medaka were raised and maintained as described previously [26]. Prior to fin-clipping, adult medaka of the Cab strain were anesthetised in 0.01% Tricaine solution (Sigma-Aldrich, A5040-25G), and all efforts were made to minimize suffering.

### sgRNA target site selection and *in vitro t*ranscription

sgRNAs were designed with CCTop as described in Stemmer et al. [3]. sgRNA for *rx2* was the same as in Stemmer et al. [3] and the *olOca2* sgRNAs were the same as in Lischik et al. [24]. The following target sites were used (PAM in brackets): *rx2 T1* (GCATTTGTCAATGGATACCC[TGG]), *olOca2 T1* (GAAACCCAGGTGGCCATTGC[AGG]), *olOca2 T2* (TTGCAGGAATCATTCTGTGT[GGG]), *drOca2 T1* (GTACAGCGACTGGTTAGTCA[TGG]). Cloning of sgRNA templates and *in vitro* transcription was performed as detailed in Stemmer, et al. [3].

### Microinjection

Medaka one-cell stage embryos were injected in the cytoplasm as previously described [3], zebrafish one-cell stage embryos were injected in the cytoplasm or yolk. Injection solutions for *oca2* targeting comprised: 150 ng/μl *Cas9* mRNA, 15 ng/μl respective sgRNA (either *olOca2 T1*, *olOca2 T2* or *drOca2 T1*) and 10 ng/μl *gfp* mRNA as injection tracer. Injected embryos were incubated at 28°C and selected for GFP expression at 1 dpf. Injection solution for *gfp-rx2* tagging comprised 150 ng/μl Cas9 mRNA, 15 ng/μl *rx2 T1* sgRNA and 5 ng/μl biotinylated PCR donor fragment [20].

### Sample preparation and imaging

Medaka and zebrafish embryos were administered to a 96 U-well microtiter plate (Nunc, Thermofisher #268152) containing either 1x embryo rearing medium or 1x zebrafish medium for automated screening/phenotyping using an ACQUIFER Imaging Machine (DITABIS AG, Pforzheim, Germany). Images were acquired in brightfield using 9 z-slices (dz = 100 μm) and a 4x Plan UW N.A. 0.06 (Nikon, Düsseldorf, Germany) to capture the centered embryo. Integration times were fixed with 100% relative white LED intensity and 50 ms exposure time. GFP channel was used on *rx2* specimens at 30% relative LED intensity and 200 ms exposure time.

### 96 U-well plate ‘hammer’

The hammer consists of 5 different components. The top plate is a 6 mm aluminum plate (material 3.3211) milled to size, drilled and countersunk to fit ISO 10642 screws and acts as a backstop for the pins. The perforated plate is made out of a 6 mm aluminum plate (material 3.3211) milled to size; the holes are drilled, reamed and countersunk on a CNC milling machine to achieve the demanded tolerances. The handle (aluminum, material 3.3211) is turned to size, drilled and threads are cut on a lathe; the OD is knurled to enhance the grip. The pins are made out of ISO 2338 (stainless steel, material 1.4301), which received a radius to fit the well ground. For the assembly, the pins are placed in a 96 well plate and properly aligned in the perforated plate and then glued in with a solvent resistant adhesive. The handle and the perforated plate are then screwed to the top plate.

### Nucleic acid extraction and precipitation using conventional protocol

Individual embryos were lysed in 1.5–2 ml tubes containing 100 μl fin-clip lysis buffer (0.4 M Tris-HCl pH 8.0, 5 mM EDTA pH 8.0, 0.15 M NaCl, 0.1% SDS in Milli-Q water) with 5 μl Proteinase K (20 mg/ml) using 1–2 ml pestles (Labortechnik Hardenberg and Eppendorf), followed by a 2-hour incubation at 60°C. 200 μl Milli-Q water was added to the embryo lysate and mixed by inversion, followed by Proteinase K inactivation at 95°C for 20 minutes. 200 μl of lysate supernatant was transferred to a clean 1.5 ml tube, and 20 μl 3M sodium acetate and 600 μl 100% ethanol absolute were added, mixed well by shaking and centrifuged at max speed for 30 minutes at 4°C. The supernatant was discarded, the pellet was air dried and resuspended in 30 μl 1x TE buffer (10 mM Tris pH 8.0, 1 mM EDTA in Milli-Q water).

### Nucleic acid extraction via filter-in-tips and locus amplification by PCR

Fin-clip lysis buffer was used for medaka, zebrafish, *A*. *thaliana*, *C*. *riparius* and *D*. *melanogaster* (0.4 M Tris-HCl pH 8.0, 5 mM EDTA pH 8.0, 0.15 M NaCl, 0.1% SDS in Milli-Q water). For *M*. *musculus* ear clip and embryonic stem cell samples 1% SDS lysis buffer was used (0.1 M Tris-HCl pH 8.0, 5 mM EDTA pH 8.0, 0.2 M NaCl, 1% SDS in Milli-Q water).

For medaka and zebrafish extraction of gDNA, see main text. The ‘hammer’ was pre-cleaned by incubation in hypochlorite solution (1:10 dilution of commercial bleach (Danklorix) reagent) for at least 15 minutes followed by 5 minutes rinsing in Milli-Q water. Plates with embryo lysates can be stored at 4°C with proper plate seal. 2 mm diameter paper puncher (Harris Uni-Core 2.0) was used on Whatman Cellulose paper (3030–917 Grade 3MM) to produce the paper discs which were transferred directly from the puncher to standard yellow 200 μl pipet tips (Steinbrenner GmbH). 50 μl PCR reaction mixes for the amplification of the desired genomic loci were prepared on ice using 1x Q5 reaction buffer, 200 μM dNTPs, 200 μM primer forward and reverse and 0.6 U/μl Q5 polymerase (New England Biolabs). 30 PCR cycles were run in all samples except for *A*. *thaliana*, *C*. *riparius* and *D*. *melanogaster* (35 cycles); annealing temperatures, extension times and primer sequences are given and PCR conditions used are listed in Table 1. Filter-in-tips were used to transfer nucleic acids from tissue lysate to the PCR mix in the following steps: Binding: pipetting sufficient tissue lysate (here 50 μl) to soak the paper disc and wait ≈10 seconds before releasing the lysate back for storage. Washing: 1 washing step by brief up and down pipetting in Milli-Q water, the higher the concentration of SDS the more washing steps are required, i.e. for 1% or higher SDS containing buffers, 4 washing steps are advised. Elution: pipetting up the PCR mix to soak the paper disc (here 50 μl), wait ≈10 seconds and release the PCR mix back into the tube for amplification. Following PCR, 10 μl PCR reaction + 2 μl 6x orange Loading Dye were loaded on 1% or 1.5% Agarose in 1xTAE gels. Gel electrophoresis was performed at 90V.

**Table 1:** List of primers and PCR conditions used. All primers used in this study are given in 5’-3’ direction. Annealing temperature, extension time and expected band size given. Note: for *gfp-rx2* two major bands are expected upon HDR-mediated integration–the wt size of non-*gfp*-integrated loci as well as the larger single-*gfp* insertion. *Rx2* tagging design is as described in Gutierrez et al., 2018 (20).

organism	gene	primers (5’– 3’ direction)	annealing temp.	extension time	band size
*Arabidopsis thaliana*	*tubulin* Lf	AGTAGTTTAAGGACCTACTTCG	60°C	14 secs	467 bp
	*tubulin* Lr	GAGCCTTACAACGCTACTCTGTCTGTC			
*Chironomous riparius*	*germ cell-less* Lf	CTTTATTAGCGTTCGGTCGTG	63°C	35 secs	1000 bp
	*germ cell-less* Lr	CCAACGTAACATTATCCTTCGC			
*Drosophila melanogaster*	*twist* Lf	CAATTTGAGCAATGGCCGGAAGGA	70°C	28 secs	931 bp
	*twist* Lr	ACTGCTGCTGCTGGTTGTTGTAGA			
*Mus* *musculus*	*trpm4* P1	GTTTGATGTCTCCTTCAGTCG	62°C	6 secs	200 bp
	*trpm4* P2	GAGTTCCTGTCCTCCTAAAGG			
	*trpm4* P3	ACCTACAGGAAACCTCGGGG			
*Danio rerio*	*drOca2* Lf	ACAGGTGCTGTATAATTGGACCAT	65°C	19 secs	619 bp
	*drOca2* Lr	AAAGAGTGGTCATAAACGGCTACT			
*Oryzias latipes*	*olOca2* Lf	GTTAAAACAGTTTCTTAAAAAGAACAGGA	62°C	21 secs	696 bp
	*olOca2* Lr	AGCAGAAGAAATGACTCAACATTTTG			
	*rx2* Lf	TGCATGTTCTGGTTGCAACG	68°C	44 secs	1457 bp
	*gfp r*	AAACGCTCGACCAGGATGGGCA			
	*rx2* Lf	TGCATGTTCTGGTTGCAACG	67°C	90 secs	1719 bp 2547 bp
	*rx2* Lr	AGGGACCATACCTGACCCTC			
	*actb* Lf	CAGCAACGACTTCGCACAAA	66°C	24 secs	797 bp
	*actb* Lr	CAGGGGCAATTCTCAGCTCA			
	*dnmtY* Lf	ACAGGTAAACCAGAAAAACTA	58°C	11 secs	375 bp
	*dnmtY* Lr	AACTAATTCATCCCCATTCC			

### PCR purification and genotyping by sequencing

PCR products were purified either using the innuPREP DOUBLEpure Kit (Analytikjena) or the QIAquick PCR Purification Kit Protocol (QIAGEN) following the manufacturer’s instructions. Purified PCRs were sent for sequencing to Eurofins Genomics (Ebersberg, Germany), the allelic distribution profiles were determined using the online toolkit TIDE with standard parameters [8].

### Genotyping by T7 Endonuclease I assay

For a fast and qualitative assay of genome editing, samples not subjected to sequencing were genotyped using the T7 Endonuclease I Assay (New England Biolabs). 10μl of the unpurified PCR reaction was directly transferred to fresh PCR tubes with 7.5 μl Milli-Q water and 2 μl NEB Buffer 2 (total 19.5 μl) and proceeded with heteroduplex formation: denature at 95°C for 5 mins, stepwise cool down from 95°C to 85°C with -2°C/sec, and stepwise cool down from 85°C to 25°C with -0.1°C/sec and subsequent digestion by adding 0.5 μl of T7EI (New England Biolabs) and incubation for 30 min at 37°C. After incubation, 10μl T7EI digests + 2 μl 6x orange Loading Dye were run on a 1% or 1.5% Agarose in 1xTAE gel at 90 V.

## Supporting information

S1 Fig 96 U-well plate mortar (the ‘hammer’) design and schematics. Construction plan and schematics of the 96 U-well plate mortar, materials and dimensions indicated.
(TIF)

S2 Fig 96-well format high-throughput SLEDGE hammer analysis. A) 96 well plate layout for high-throughput genotyping. CRISPR/Cas9 mediated (green wells) knock-out of *oca2* locus with three individual sgRNAs: *olOca2 T1* (columns 1–3), *olOca2* T2 (columns 4–6), *drOca2 T1* (columns 7–9). HDR/donor mediated integration of *gfp* in frame with *rx2* locus (columns 10–12). Medium control (blue wells) and uninjected wildtype specimens (red wells) included for control. B) Successful rapid extraction/transfer of gDNA using filter-in-tips evident by *oca2* locus PCR amplification of injected and uninjected specimens. Larger random indel formation can yield extra bands (black asterisks). C) T7EI assay of locus amplification in B reveals specificity of gDNA transfer method by T7EI digestion of heteroduplexes (cut bands) in *oca2* crispants but not wildtype embryos. D) *rx2* locus PCR amplification of injected and uninjected specimens. Note: non-*gfp*-integrated locus band (black arrowhead, 1719 bp) and single precise *gfp* integration (green asterisks, 2547 bp) evident by band-size. Additional bands stem from NHEJ-events. E) *gfp-rx2* specific bands correlate with embryos expressing GFP in retinae. All single-copy HDR-mediated *gfp* integration events in D could as well be verified by band size (953 bp) here. In addition, some donors underwent NHEJ (red asterisk, ≈1400 bp) or most probable concatenation events (yellow asterisks).
(TIF)
