# Supplementary figures and images for: Swift Large-scale Examination of Directed Genome Editing
Source: PLoS One. 2019 Mar 5;14(3):e0213317. doi: 10.1371/journal.pone.0213317 (PMC6400387; doi:10.1371/journal.pone.0213317)

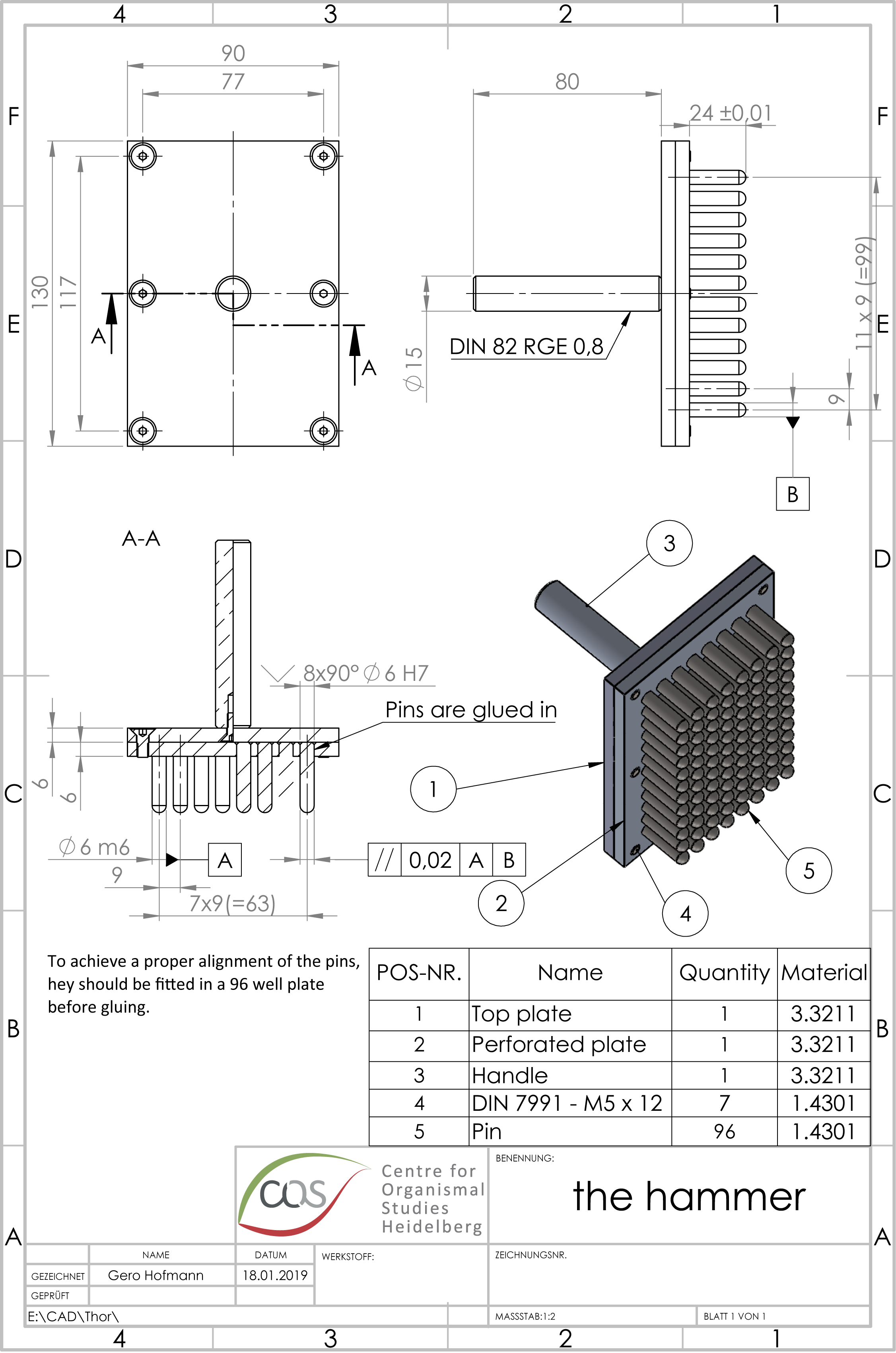

Supplement: S1 Fig — Construction plan and schematics of the 96 U-well plate mortar, materials and dimensions indicated. (TIF) [file pone.0213317.s001.tif]

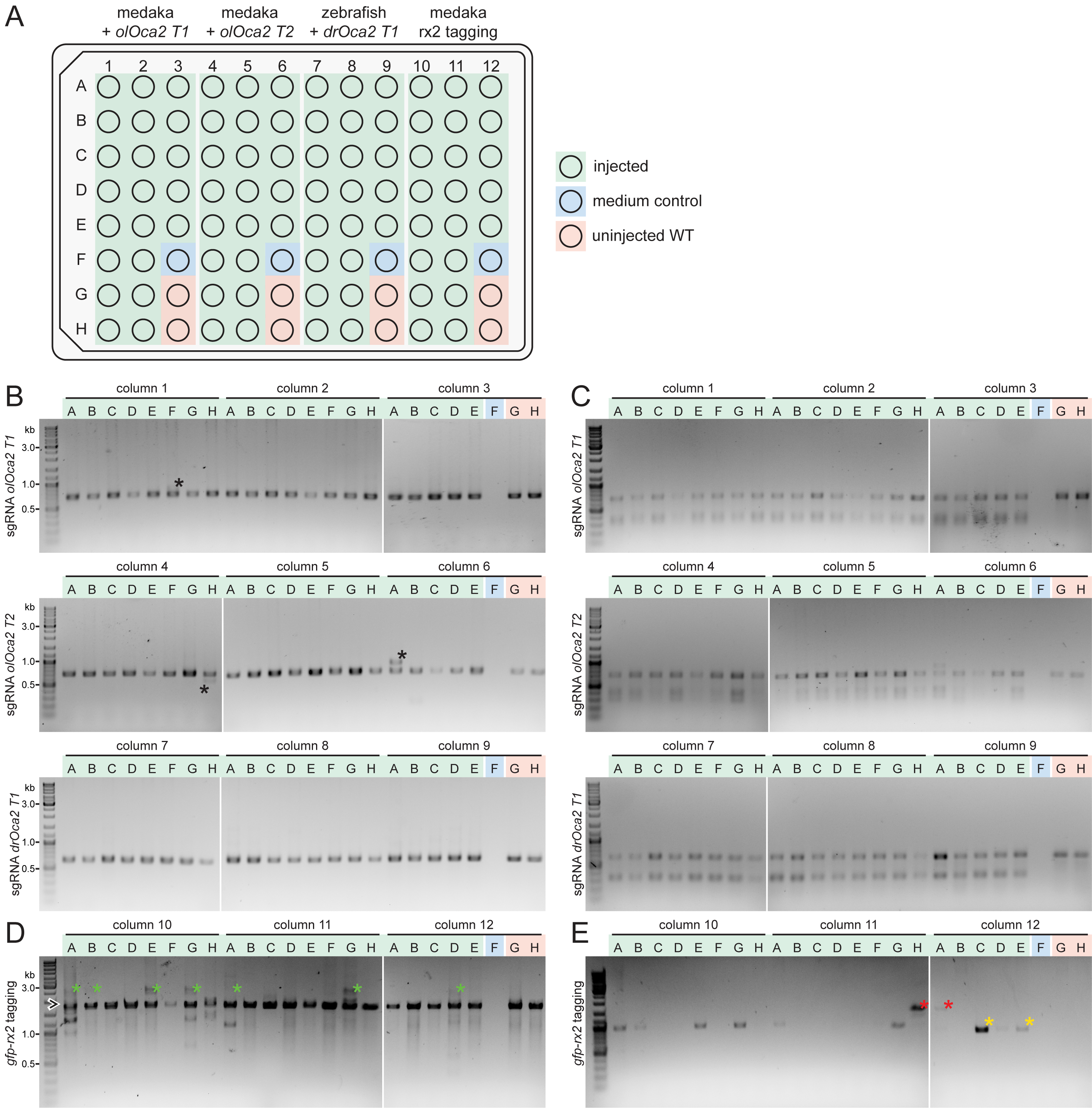

Supplement: S2 Fig — A) 96 well plate layout for high-throughput genotyping. CRISPR/Cas9 mediated (green wells) knock-out of oca2 locus with three individual sgRNAs: olOca2 T1 (columns 1–3), olOca2 T2 (columns 4–6), drOca2 T1 (columns 7–9). HDR/donor mediated integration of gfp in frame with rx2 locus (columns 10–12). Medium control (blue wells) and uninjected wildtype specimens (red wells) included for control. B) Successful rapid extraction/transfer of gDNA using filter-in-tips evident by oca2 locus PCR amplification of injected and uninjected specimens. Larger random indel formation can yield extra bands (black asterisks). C) T7EI assay of locus amplification in B reveals specificity of gDNA transfer method by T7EI digestion of heteroduplexes (cut bands) in oca2 crispants but not wildtype embryos. D) rx2 locus PCR amplification of injected and uninjected specimens. Note: non-gfp-integrated locus band (black arrowhead, 1719 bp) and single precise gfp integration (green asterisks, 2547 bp) evident by band-size. Additional bands stem from NHEJ-events. E) gfp-rx2 specific bands correlate with embryos expressing GFP in retinae. All single-copy HDR-mediated gfp integration events in D could as well be verified by band size (953 bp) here. In addition, some donors underwent NHEJ (red asterisk, ≈1400 bp) or most probable concatenation events (yellow asterisks). (TIF) [file pone.0213317.s002.tif]
